# The Nervous Diseases of Women

**Published:** 1874-06

**Authors:** Allan McLane Hamilton

**Affiliations:** Late Physician in charge of the New York. State Hospital for Disease of the Nervous System


BUFFALO

### Medical and Surgical Journal.

VOL XIII. JUNE, 1874. No. 11.

### Original Communications.

———:o:———

ART. I.—*The Nervous Diseases of Women.* By ALLAN McLANE HAMILTON, M. D., late Physician in charge of the New York. State Hospital for Disease of the Nervous System.

The intimate relationship between the female organs of generation and the nervous system, both by the great dependence on each other, and the sympathy with other systems of the body, furnishes us when these organs are deranged, with a large field for observation and clinical research.

Though the female at all times of her life, is subject to nervous diseases, either local, or secondarily through the transmission of irritation to the cerebro-spinal axis; there are certain periods when this liability is the greatest—these are, with the beginning of menstruation, at marriage and parturition, and at the menopause. At these times the womans body undergoes an unusual revolution. The pelvic organs are hyperæmic, and there are various strong impressions made upon the sympathetic and cerebro-spinal systems. These, if not properly braced and nourished, are subjected to severe strains, and are irritated to such a degree that unless the other parts of the body are up to a normal standard, a number of serious nerve changes are produced.

These affections may be either of short duration or may last for a very long period, if there be a temporary morbid irritation sent to the nerve centers, or one transmitted long enough to change the condition of the central nerve cells. If this abnormal irritation is transmitted for a long period, and actual pathological changes take place, the chance of cure is poor.

I have known an anteflexion of the uterus to produce an epilepsy which has defied the most persistent treatment even after the malposition of this organ had been overcome.

If the nervous disease begins with the first menstruations, the prognosis is better than if it comes on later in life, from a cause long unrecognized. The condition of the patient in youth, enables us to treat her with more success than later in life, when the vital powers have been shattered and the nerve centers changed.

The nervous diseases incident to woman and specially connected with the generative organs, may be said to be local or primary, and reflex or secondary.

The local diseases include the various neuralgiæ and hyperæsthesiæ, including certain symptoms of local derangement, not clearly nervous in their expression; for instance menorrhagia where there is paresis of the vaso motor nerves.

The secondary disorders which I have spoken of, may consist in the beginning of functional derangements of circulation expressed in transitory attacks of cerebral congestion, or anæmia, and characterized by headache, neuralgia, hysteria, temporary insanity, or choreaform convulsions.

More severe conditions may ensue; permanent melancholia, deep seated neuralgia, epilepsy and apoplexy, and various kindred diseases.

There are many simple conditions of deranged sensibility which usually have the vagina as their seat. These may be vaginismus, vaginitis when accompanied with highly hyperæsthetic tubercules in its surface and about the vulva. In a woman of nervous predisposition a vaginitis may often produce a reflex condition of great importance. Hysteria or various mental disorders, and neuralgia.

The local uterine neuroses may be spoken of as dysmenorrhœa with its accompanying neuralgia. Sudden congestion of the uterus—or violent irritation of this organ in a congested state, is followed by central nervous disturbance oftentimes of great intensity. These conditions may be excited at the time of marriage when the peripheral ends of the nerve supplying those organs, may receive a shock, transmitted afterwards to the cord and brain. A rudimentary uterus and undeveloped state of the parts of generation predisposed to many morbid nervous changes when any sudden excitation is brought to bear upon them; a case in point suggests itself to me.

M. S. aet., 34. The patient was unmarried until six months ago, when she fell deeply in love with a man much younger, and full of passion. The patient herself, is of a strong nervous temperament and with a hereditary predisposition. She had suffered from amerrorrhœa and neuralgia before marriage.

A week after her marriage, she showed signs of discontent, but to some degree enjoyed intercourse. She became melancholy however at the end of a month, and refused to answer questions—she was preoccupied and sat by herself from the rest of her family, abstracted and gloomy. She next had hallucinations and delusions, believing that she was the victim of her husbands persecution, while in reality he was consic erate and kind. Her hands and face were dusky and cold, and her eyes expressionless. Her husband took her to a country practitioner who failed to see any cause for the mental change. As she advanced she became sleepless and restless, and the narcotics given by her physician failed the procure sleep.

She was brought to me four months after the marriage, and after carefully examining her, I found a very small rudimentary uterus no larger than a childs before puberty. There was a swollen condition of the parts in the vicinity. Her friends were directed to keep her apart from her husband, and a course of treatment advised.

In this case the shock to these parts by coition was clearly the cause of the melancholia.

There are numerous instances where slighter causes will produce equally severe symptoms of irritation transmitted to the nerve centres. I found in one case of general nervous disturbance, hysteria and great neurasthenia in the person of a married woman, that a small round tumor richly supplied with sensitive nerves and exquisitively painful to the touch, was located on the vulva, and that every effort of her husband at coition was attended with a perfect vaginismus. These conditions disappeared when the growth was removed.

Different malpositions of the uterus are productive of deep seated diseases, the etiology of which appears obscure unless the physician is on the lookout.

I recently cited a case of this kind in an article published in the *New York Medical Record*, Dec., 15th, 1873. The patient had a flexion of the uterus, which was followed by attacks of epilepsy. I have seen other of the same class. In this case the patient recovered to a great extent in a few weeks.

Hysteria among women is certainly connected in most instances with some uterine or ovarian disease. The following is a case of comatose hysteria such as Dr. Hanfield Jones describes in his work on "Functional Nervous Disorders." It shows how slight a condition may produce a grave form of disease.

———— female aet. 18. The patient was picked up by a policeman at three o'clock in the morning, in an almost nude condition, and taken to the Brooklyn City Hospital. She was unconscious at the time, and remained so for several days, six in all I think. On admission she was convulsed with tonic spasms of nearly all the voluntary muscles, with occasional clonic spasms of some of the muscles of the upper extremities. There was moderate opisthotonus, and the teeth were set, and eyeballs rolled upwards. There would be occasional nystagmus, and she would moan and roll her head sideways. Her abdomen was tympanitic, and large stimulating enemata were thrown into the rectum to bring away masses of feces, and scyballæ. Hydrate of chloral was given in large doses, and at the end of the second day she opened her eyes which had been closed meanwhile, and looked very rationally. Her muscles gradually relaxed, and she sank into a troubled sleep with occasional muttering.

We made an examination, and discovered a large chancriod on the cervix. The vaginal walls were congested and tumefied. Warm vaginal injections were applied and the menses established.

Her history was a peculiar one. She had escaped from one of the Blackwell's Island Hospitals probably in an irresponsible condition, and had wandered to the spot where she was picked up.

There are cases of hysteria in which prolapsus has been the exciting cause, or when simple pruritus vulvæ dependent upon masses of smegma within the labia, have acted as foci of irritation and produced reflex impressions.

Of the ovarian neuroses, neuralgia is most common. It is usually found with enlargement of the uterus. When there is enlargement or engorgement of the uterus, the form is commonly sciatica. There is local hyperæsthesia in many cases, and the neuralgic pains may be paroxysmal or steady. They are always aggravated by walking or standing.

A number of ill defined symptoms come under the head of dysæsthesia; fleeting pains about the pelvis and morbid feelings, without any assignable cause, very readily yield to treatment, as they are due to functional causes.

Women who suffer from want of sleep, irritability and general depression, can generally lay the cause of the trouble to a disturbance of the functions of the ovaries or uterus. The sympathetic system is in such close connection with the generative and digestive systems, that with any derangment of one, the other is sure to be affected. Many cases of sleeplessness laid to other causes have their starting point in the pelvis.

